# SARS-CoV-2 environmental contamination from hospitalised patients with COVID-19 receiving aerosol-generating procedures

**DOI:** 10.1136/thoraxjnl-2021-218035

**Published:** 2021-11-04

**Authors:** Rebecca L Winslow, Jie Zhou, Ella F Windle, Intesar Nur, Ranjit Lall, Chen Ji, Jonathan Edward Millar, Paul M Dark, Jay Naisbitt, Anita Simonds, Jake Dunning, Wendy Barclay, John Kenneth Baillie, Gavin D Perkins, Malcolm Gracie Semple, Daniel Francis McAuley, Christopher A Green

**Affiliations:** 1 Department of Infectious Diseases and Tropical Medicine, University Hospitals Birmingham NHS Foundation Trust, Birmingham, UK; 2 The Epidemiology and Public Health Group (EPHG), Division of Population Health, Health Services Research and Primary Care, University of Manchester, Manchester, UK; 3 Department of Infectious Diseases, Imperial College London, London, UK; 4 College of Medical and Dental Sciences, University of Birmingham, Birmingham, UK; 5 Warwick Clinical Trials Unit, Warwick Medical School, University of Warwick, Coventry, UK; 6 Roslin Institute, University of Edinburgh, Midlothian, UK; 7 NIHR Manchester Biomedical Research Centre, Division of Infection, Immunity and Respiratory Medicine, Faculty of Biology, Medicine and Health, University of Manchester, Manchester, UK; 8 Critical Care Unit, Northern Care Alliance NHS Group, Salford Royal Hospital, Greater Manchester, UK; 9 Lung Division, Royal Brompton and Harefield NHS Foundation Trust, London, UK; 10 Faculty of Medicine, Imperial College London, London, UK; 11 Department of Critical Care Medicine, University Hospitals Birmingham NHS Foundation Trust, Birmingham Heartlands Hospital, Birmingham, UK; 12 NIHR Health Protection Research Unit in Emerging and Zoonotic Infections, Institute of Infection, Veterinary and Ecological Sciences, Faculty of Health and Life Sciences, University of Liverpool, Liverpool, UK; 13 Department of Respiratory Medicine, Alder Hey Children's Hospital, Liverpool, UK; 14 Wellcome-Wolfson Institute for Experimental Medicine, Queen's University Belfast, Belfast, UK; 15 Regional Intensive Care Unit, Royal Victoria Hospital, Belfast, UK; 16 Institute of Microbiology and Infection, University of Birmingham, Birmingham, UK

**Keywords:** COVID-19, non invasive ventilation

## Abstract

**Background:**

Continuous positive airways pressure (CPAP) and high-flow nasal oxygen (HFNO) are considered ‘aerosol-generating procedures’ in the treatment of COVID-19.

**Objective:**

To measure air and surface environmental contamination with SARS-CoV-2 virus when CPAP and HFNO are used, compared with supplemental oxygen, to investigate the potential risks of viral transmission to healthcare workers and patients.

**Methods:**

30 hospitalised patients with COVID-19 requiring supplemental oxygen, with a fraction of inspired oxygen ≥0.4 to maintain oxygen saturation ≥94%, were prospectively enrolled into an observational environmental sampling study. Participants received either supplemental oxygen, CPAP or HFNO (n=10 in each group). A nasopharyngeal swab, three air and three surface samples were collected from each participant and the clinical environment. Real-time quantitative polymerase chain reaction analyses were performed for viral and human RNA, and positive/suspected-positive samples were cultured for the presence of biologically viable virus.

**Results:**

Overall 21/30 (70%) participants tested positive for SARS-CoV-2 RNA in the nasopharynx. In contrast, only 4/90 (4%) and 6/90 (7%) of all air and surface samples tested positive (positive for E and ORF1a) for viral RNA respectively, although there were an additional 10 suspected-positive samples in both air and surfaces samples (positive for E or ORF1a). CPAP/HFNO use or coughing was not associated with significantly more environmental contamination than supplemental oxygen use. Only one nasopharyngeal sample was culture positive.

**Conclusions:**

The use of CPAP and HFNO to treat moderate/severe COVID-19 did not appear to be associated with substantially higher levels of air or surface viral contamination in the immediate care environment, compared with the use of supplemental oxygen.

**Key messages**

**What is the key question?**

- Do hospitalised patients with COVID-19 receiving treatment with continuous positive airways pressure (CPAP) and high-flow nasal oxygen (HFNO) present a significant added risk of viral contamination to the surrounding environment used by healthcare workers?

**What is the bottom line?**

- The use of CPAP or HFNO to treat moderate/severe COVID-19 did not produce significant additional air or surface viral contamination compared with supplemental oxygen.

**Why read on?**

- The evolving evidence from hospitalised patients with SARS-CoV-2 infection and the risks of occupational/nosocomial exposure should prompt an evidence-based reassessment of infection prevention and control measures for non-invasive respiratory support treatments that are currently considered ‘aerosol generating procedures.’

## Introduction

Severe acute respiratory syndrome coronavirus 2 (SARS-CoV-2) is a novel β coronavirus that has led to the global pandemic of coronavirus disease 2019 (COVID-19), as declared by the WHO on 11 March 2020. Transmission is by close contact, droplets (>5–10 µm diameter) that deposit closer to their source, and airborne inhalation of aerosols (<5 µm diameter) that are suspended in the air for longer, travel further and have the potential to reach the alveolar region of the lung. Airborne transmission has historically been associated with the use of aerosol-generating procedures.^1 2^

UK data from 2020 estimated that 17% of all emergency COVID-19 admissions required respiratory support in high-dependency or intensive care settings, which included the use of non-invasive respiratory support and mechanical ventilation for moderate/severe cases (16% and 10% of all admissions, respectively).^3^ Types of non-invasive respiratory support commonly include the use of continuous positive airway pressure (CPAP) and high-flow nasal oxygen (HFNO) devices which have been associated with reductions in mortality and progression to intubation for hypoxaemic respiratory failure in some studies.^4 5^ Their effectiveness in the treatment of COVID-19 is currently under evaluation in randomised controlled trials. Both are widely designated as aerosol-generating procedures and necessitate additional airborne precautions, including cohorting of patients and the use of FFP3 masks for healthcare workers (HCWs) to mitigate the risk of aerosol transmission.^6 7^ However, this is based on weak evidence from the SARS-CoV-1 outbreak and may delay or restrict patient access to these therapies.^8^ Nosocomial transmission from earlier coronavirus outbreaks (SARS-CoV-1 and MERS-CoV) were reported as up to 80% and 40% for patients and HCWs, respectively,^9^ and recent studies suggest that HCWs are a population with a substantial burden from COVID-19, particularly in non-intensive care settings where airborne precautions are less frequently used.^10 11^

SARS-CoV-2 environmental contamination has been widely found in multiple studies; however, very few have specifically evaluated the impact of CPAP and/or HFNO, or have found biologically viable virus that proves a transmission risk to HCWs.^12–20^ Other studies in this field include aerosol-generation studies that have mainly used patient simulators or healthy volunteers.^21–24^ Here we report our observations from sampling the clinical environment of patients with COVID-19 undergoing CPAP and HFNO, compared with the use of supplemental oxygen, to better understand the risks of airborne and fomite SARS-CoV-2 contamination and exposure to HCWs.

## Materials and methods

### Study design, participants and setting

This study was a prospective observational study of environmental viral contamination from hospital admissions with COVID-19 as part of the International Severe Acute Respiratory and emerging Infections Consortium (ISARIC) WHO Clinical Characterisation Protocol UK (CCP-UK, www.isaric4c.net). It was performed across three UK hospitals at University Hospitals Birmingham NHS Foundation Trust and study participants were NHS patients co-enrolled (or who were eligible to be co-enrolled) into ISARIC WHO CCP-UK and the RECOVERY-Respiratory Support trial.^25^ Participant inclusion criteria included having suspected or confirmed SARS-CoV-2 infection with hypoxaemia (defined as requiring supplemental oxygen with a fraction of inspired oxygen ≥0.4 to maintain oxygen saturations ≥94%) and suitable for CPAP or HFNO. Participants were enrolled into one of three groups (n=10 per group); CPAP, HFNO or supplemental oxygen, within 5 days of starting treatment. Recruitment was opportunistic and written informed consent was obtained before any study procedures were undertaken. The machines used to deliver CPAP were either a Philips Respironics Trilogy, V60 using ResMed AcuCare masks with heat moisture exchange filter, or the University College London Ventura system with viral filters, and all were capable of flow rates from 15 to 60 L/min. HFNO was delivered by a Fisher and Paykel Airvo2 system using Optiflow nasal cannulae (OPT944) with a typical flow rate of between 50 and 60 L/min. Participants received supplemental oxygen via a Venturi face mask with a maximal flow of 15 L/min. The flow rate, fraction of inspired oxygen and positive end expiratory pressures were set according to clinical need.

### Data and sample collection

Environmental samples were taken from the care setting of each participant, which varied according to clinical and operational needs. Basic demographic and clinical data were collected with samples in a single visit that lasted up to 60 min. Room temperature, humidity and carbon dioxide levels were recorded using a Therm M2000C air quality monitor. Nasopharyngeal samples were collected using a mid-turbinate flocked swab in accordance with standard operating procedures and stored in viral transport medium (VTM). Air samples were collected using a Coriolis micro air sampler (Bertin Technologies, France) that uses liquid cyclonic technology able to collect particles from 0.5 µm in diameter.^16^ The device inlet was aligned to the mouth of the participant at a distance of 50 cm, and sampled the air on three occasions, each for 10 min at a flow rate of 300 L/min (total 9 m^3^ air). The first air sample was collected with the participant at rest with supplemental oxygen only. Where the participant was unable to tolerate removal of CPAP/HFNO for the first sample, this was collected on CPAP/HFNO in order to keep the sampling period consistent for all participants. The next air sample was with CPAP/HFNO in place for a minimum of 5 min (or supplemental oxygen) and the third air sample involved the addition of voluntary coughing every 2 min. All surface samples were taken from within 2 m of the participant and used sterile flocked swabs (Coplan, US) pre-moistened with VTM to swab 25 cm^2^ from the floor, the bed table and a high-object (above participant head height such as a light fitting), in accordance with WHO sampling guidance.^26^ All swabs were placed into 1 mL of VTM. All samples were stored on ice for less than 2 hours before being stored at −80°C and later transported in accordance with UN3373 using chilled biotherm containers that maintained storage temperature at 4–6°C for laboratory analysis at Imperial College London.

### Detection and quantification of human and SARS-CoV-2 viral RNA by real-time polymerase chain reaction and viral cultures

Laboratory analyses were performed blinded to study group. Viral RNA detection and quantification was performed using quantitative real-time reverse transcription polymerase chain reaction (RT-qPCR), as described elsewhere.^16^ In summary, samples were extracted from 200 µL of the VTM using the QIAsymphony SP (Qiagen, Germany) instrument according to the manufacturer’s instructions and SARS-CoV-2 viral RNA was detected using AgPath-ID One-Step RT-PCR Reagents (Life Technologies) with specific primers and probes targeting the envelope (E)^27^ and ORF1a genes.^28^ A standard curve with six serial dilutions of 1×10^5^ – 1×10° copies/μL E gene was included in each run of the RT-qPCR. A sample was defined as positive for viral RNA if both E and ORF1a RT-qPCR assays gave cycle time (Ct) values <45. A Ct value <45 for only one of these viral gene targets was considered a suspected-positive result. A one-step RT-qPCR assay targeting human RNaseP was used to indicate human biological material in nasopharyngeal and surface swabs.^29^ Human biological material in air samples was quantified by a one-step RT-qPCR assay targeting human 18 s rRNA (18 s rRNA_Forward 5’-GGTAACCCGTTGAACCCCAT-3’, 18 s rRNA_Reverse 5’-CAACGCAAGCTTATGACCCG-3’, 18 s rRNA_Probe 5’-FAM-GTGATGGGGATCGGGGATTG-BHQ1-3’). A sample was defined as positive for human RNA if the Ct value was <45. Vero E6 (African Green monkey kidney) cells expressing angiotensin-converting enzyme 2 (ACE2) and transmembrane serine protease 2 (TMPRSS2) were used to culture virus from any positive/suspected-positive viral RNA sample. Vero cells were maintained in Dulbecco's modified Eagle's medium, supplemented with heat-inactivated fetal bovine serum (10%) and penicillin/streptomycin (10 000 IU/mL and 10 000 µg/mL). For virus isolation, 200 µL of samples were added to 24-well plates. On day 0 and after 5–7 days the cell supernatants were collected, and RT-qPCR used to detect SARS-CoV-2 RNA as described above. Samples with at least one log increase in copy numbers for the E gene (reduced Ct values relative to the original samples) after 5–7 days' propagation in cells compared with the starting value were considered positive by viral culture.^30^

### Statistical analyses

This was an exploratory study intended to be descriptive, no formalised sample size was calculated, and a sample size of 30 was used to allow for some stability in the estimates (mean and SD) of the outcomes.^31^ Analysis of variance (ANOVA/Kruskal-Wallis, as appropriate) was used to provide an overall comparison of the three groups, and significant variations were further explored by pairwise comparisons (unpaired t-tests against the supplemental oxygen group). All statistical tests were two-tailed and a p value <0.05 was considered statistically significant. The statistical tests were performed in SAS and Prism 7 (GraphPad Inc, USA). Statistically significant differences should be interpreted with caution as the study was not powered to detect differences in the treatment arms.

## Results

Thirty-two eligible patients were invited to take part and two declined to participate (one CPAP and one supplemental oxygen). Samples from the 30 enrolled participants were collected between 11 December 2020 and 19 February 2021, when the dominant variant was likely to have been B.1.1.7. The study population demographics, clinical characterisation of COVID-19 disease, and environmental conditions of the care provided to them are presented in table 1. All participants required oxygen support on admission and started dexamethasone the same day. Participant demographics were comparable across the study groups. Participants from the HFNO group were sampled significantly later in their illness than those receiving supplemental oxygen (mean 16-days, 95% CI 13 to 19, vs mean 9 days, 95% CI 5 to 13, from symptom onset, respectively) and participants receiving supplemental oxygen were sampled significantly earlier into their hospital stay (median 1 day, IQR 0–2, compared with CPAP median of 4.5 days, IQR 2–6, and HFNO median of 3 days, IQR 2–6) (online supplemental figure 1). Similar proportions of patients in each study group were cared for in cohorted areas or side room settings. Participants receiving CPAP/HFNO were more commonly accommodated in negative-pressure rooms. Compared with patients receiving supplemental oxygen, the room air recordings measured significantly lower temperatures for HFNO, with lower CO_2_ content and humidity for CPAP (online supplemental figure 2).

10.1136/thoraxjnl-2021-218035.supp1 Supplementary data

**Table 1:** Baseline clinical characteristics of study participants and the environment of care provision.

	All	SOC	CPAP	HFNO	Statistically significant differences
**Number of participants**	30	10	10	10	–
**Male (n)**	17	6	5	6	–
**Mean age** (95% CI) (min max)	56(52 to 60) (35–75)	54(47 to 61) (35–74)	60(52 to 68) (44–75)	53(45 to 61) (39–68)	p=NS (ANOVA)
**Ethnicity**					
Asian – Pakistani (n)	10	2	6	2	–
White - British (n)	8	4	0	4	–
Not given (n)	4	1	3	0	–
Asian - Indian (n)	3	0	0	3	–
Asian - other (n)	2	1	0	1	–
White - other (n)	1	1	0	0	–
Caribbean (n)	1	1	0	0	–
Mixed – White and Caribbean (n)	1	0	1	0	–
**Mean number of days of illness at time of hospital admission** (95% CI) (min–max)	9(8 to 11) (0–17)	8(5 to 11) (2–15)	8(6 to 11)) (3–12)	11(8 to 14) (0–15)	p=NS (ANOVA)
**Mean number of days of illness at time of sampling** (95% CI) (min–max)	12(10 to 14) (3–25)	9(5 to 13) (3–18)	13(9 to 16) (6–24)	16(13 to 19) (11–25)	p=0.02 (ANOVA) SOC vs CPAP p=NS (unpaired t-test) SOC vs HFNO p<0.01 (unpaired t-test)
**Median number of days in hospital at the time of sampling** (IQR) (min–max)	2(1–5) (0–14)	1 (0–2) (0–3)	4.5(2–6) (1–9)	3(2–6) (2–14)	p<0.01 (Kruskal-Wallis) SOC vs CPAP p<0.01 (Mann-Whitney) SOC vs HFNO p<0.01 (Mann-Whitney)
**Mean number of days CPAP/HFNO at time of sampling** (95% CI) (min–max)	N/A	N/A	2.4 [1.5–3.3] (1-4)	1.8 [1.0–2.6] (0–3)	–
**Median FiO_2_ at time of sampling** (IQR) (min-max)	56(40 to 73) (35–98)	59(40 to 65) (40–98)	48(40 to 62) (40–80)	63(40 to 91) (35–98)	p=NS (Kruskal-Wallis)
**Mean SpO_2_ at time of sampling** (95% CI mean) (min–max)	94(93 to 95) (92–99)	95(93 to 96) (92–99)	94(93 to 95) (92–96)	94(93 to 96) (92–98)	p=NS (ANOVA)
**Room type**					
Open bay/cohort area	12	4	4	4	–
Side room – ambient pressure	8	5	0	3	–
Side room – negative pressure	7	0	6	1	–
Side room – natural airflow	3	1	0	2	–
**Estimated air changes per hour**					
10	15	6	6	6	–
4–6	10	4	4	2	–
4	2	0	0	2	–
**Mean room air temperature (°C) at time of sampling** (95% CI) (min–max)	21.9(21 to 23) (18.0–25.0)	23.2(22 to 24) (20.0–25.0)	21.9(21 to 23) (19.0–24.0)	20.7(19 to 22) (18.0–23.0)	p=0.01 (ANOVA) SOC vs CPAP p=NS (unpaired t-test) SOC vs HFNO p<0.01 (unpaired t-test)
**Median room air CO_2_ content (ppm) at time of sampling** (IQR) (min–max)	574.5(500–808) (419–1548)	672.5(530–774) (459–1548)	502.0(448–582) (419-618)	915.0 (459–1303) (506–1460)	p=0.01 (Kruskal-Wallis) SOC vs CPAP p=0.02 (Mann-Whitney) SOC vs HFNO p=NS (Mann-Whitney)
**Mean room air humidity (%) at time of sampling** (95% CI mean) (min–max)	37.6(34 to 41)(22.0–58.0)	37.5(32 to 43)(23.0–41.0)	30.4(26 to 35)(23.0–41.0)	44.8(37 to 53)(26.0–58.0)	p<0.01 (ANOVA) SOC vs CPAP p=0.03 (unpaired t-test) SOC vs HFNO p=NS (unpaired t-test)
**Receiving humidified oxygen (n)**	15	6	2	7	–
**CPAP full face mask (unvented) (n)**	N/A	N/A	8	N/A	–
**CPAP partial face mask (vented) (n)**	N/A	N/A	2	N/A	–

A total of 30 participants with moderate/severe COVID-19 were enrolled into the study. Paired t-tests were post-hoc analysis of differences between SOC and CPAP/HFNO study groups only.

ANOVA, analysis of variance; CPAP, continuous positive airway pressure; FiO_2_, fraction of inspired oxygen; HFNO, high-flow nasal oxygen; N/A, not applicable; NS, not significant; SOC, supplemental oxygen care; SpO_2_, oxygen saturation.

### Participants had detectable viral RNA in the nasopharynx at the time of environmental sampling

Overall 21/30 (70%) of participants tested positive for SARS-CoV-2 RNA in the nasopharynx at the time of environmental sampling (table 2). An additional participant was a suspected-positive case and all study participants tested positive on PCR testing either in the community or on admission to hospital (data not shown). Ct values, as an inversely related measure or estimate of genetic quantity (meaning low Ct values are indicative of greater target gene quantity in the sample), were also analysed for all samples. For positive nasopharyngeal samples, the mean Ct value was 29.2 (95% CI 27 to 32) and were comparable across different study groups with high correlation between the Ct value for each gene (r^2^=0.95). There were no correlations between the Ct values of any viral genes and the duration of illness/hospital stay (online supplemental figure 3).

**Table 2:** The frequencies of SARS-CoV-2 RNA positive, suspected-positive and negative samples.

	All	SOC	CPAP	HFNO
**Number of participants**	30	10	10	10
**Nasopharyngeal samples**				
Number positive/suspected-positive/negative (overall % positive or suspected-positive) Mean Ct value (95% CI) for lowest Ct value for E or ORF1a only	21/1/8 (73%) 29.2(27 to 32)	8/1/1 (90%) 29.8(26 to 34)	8/0/2 (80%) 31.2(27 to 35)	5/0/5 (50%) 24.9(18 to 32)
**Overall for air samples** Number positive/suspected-positive/negative (overall % positive or suspected-positive)	4/10/76 (16%)	1/4/25 (17%)	0/2/28 (7%)	3/4/23 (23%)
**Air samples collected with participant breathing normally (SOC or CPAP/HFNO off**) Number positive/suspected-positive/negative (overall % positive or suspected-positive) Mean Ct value (95% CI) for lowest Ct value for E or ORF1a only	2/4/24 (20%) 38.2(35 to 41)	1/2/7 (30%) 39.7(32 to 48)	0/1/9 (10%) 37.3(-)	1/1/8 (20%) 36.3(7 to 66)
**Air samples collected with participant breathing normally (SOC or CPAP/HFNO on**) Number positive/suspected-positive/negative (overall % positive or suspected-positive) Mean Ct value (95% CI) for lowest Ct value for E or ORF1a only	1/3/26 (13%) 39.0(34 to 44)	0/1/9 (10%) 37.4(-)	0/0/10 (0%) -(-)	1/2/7 (30%) 39.6(31 to 48)
**Air samples collected with participant coughing every 2 min (SOC or CPAP/HFNO on**) Number positive/suspected-positive/negative (overall % positive or suspected-positive) Mean Ct value (95% CI)for lowest Ct value for E or ORF1a only	1/3/26 (13%) 38.6(35 to 42)	0/1/9 (10%) 39.9(-)	0/1/9 (10%) 39.9(-)	1/1/8 (20%) 37.5(13 to 63)
**Overall for surface samples** Number positive/suspected-positive/negative (overall % positive or suspected-positive)	6/10/74 (18%)	1/4/25 (17%)	3/3/24 (20%)	2/3/25 (17%)
**Floor surfaces** Number positive/suspected-positive/negative (overall % positive or suspected-positive) Mean Ct value (95% CI) for lowest Ct value for E or ORF1a only	5/4/21 (30%) 37.3(36 to 48)	1/1/8 (20%) 35.8(18 to 54)	3/1/6 (40%) 36.8(36 to 38)	1/2/7 (30%) 38.9(37 to 41)
**Table surfaces** Number positive/suspected-positive/negative (overall % positive or suspected-positive) Mean Ct value (95% CI) for lowest Ct value for E or ORF1a only	0/3/27 (10%) 39.0(36 to 42)	0/2/8 (20%) 38.5(30 to 47)	0/0/10 (0%) -(-)	0/1/9 (10%) 40.0(-)
**High-object surfaces** Number positive/suspected-positive/negative (overall % positive or suspected-positive) Mean Ct value (95% CI) for lowest Ct value for E or ORF1a only	1/3/26 (13%) 37.8(35 to 41)	0/1/9 (10%) 39.4(-)	0/2/8 (20%) 38.4(32 to 45)	1/0/9 (10%) 34.8(-)

A Ct value<45 for both the SARS-CoV-2 E gene and ORF1a gene was considered a positive result. A suspected positive result was recorded when only E or ORF1a Ct values were <45. A negative result was recorded when both E and ORF1a Ct values were ≥45. Nasopharyngeal samples were collected according to local standard operating procedures and air samples and surfaces samples were collected per participant in accordance with the clinical study plan. There were no statistically significant differences in the Ct values of viral RNA in nasopharyngeal samples between study groups (p=NS, two-way ANOVA), and no statistically significant differences in the proportion of negative samples in each air and surface sample across the study groups (p=NS, Fisher’s exact test). Alternative statistical tables are available in the online supplemental material.

ANOVA, analysis of variance; CPAP, continuous positive airway pressure; Ct, cycle time; HFNO, high-flow nasal oxygen; SOC, supplemental oxygen care.

### Low levels of viral RNA in air samples, regardless of whether CPAP or HFNO was in use or if the participant was coughing

**Figure 1:**
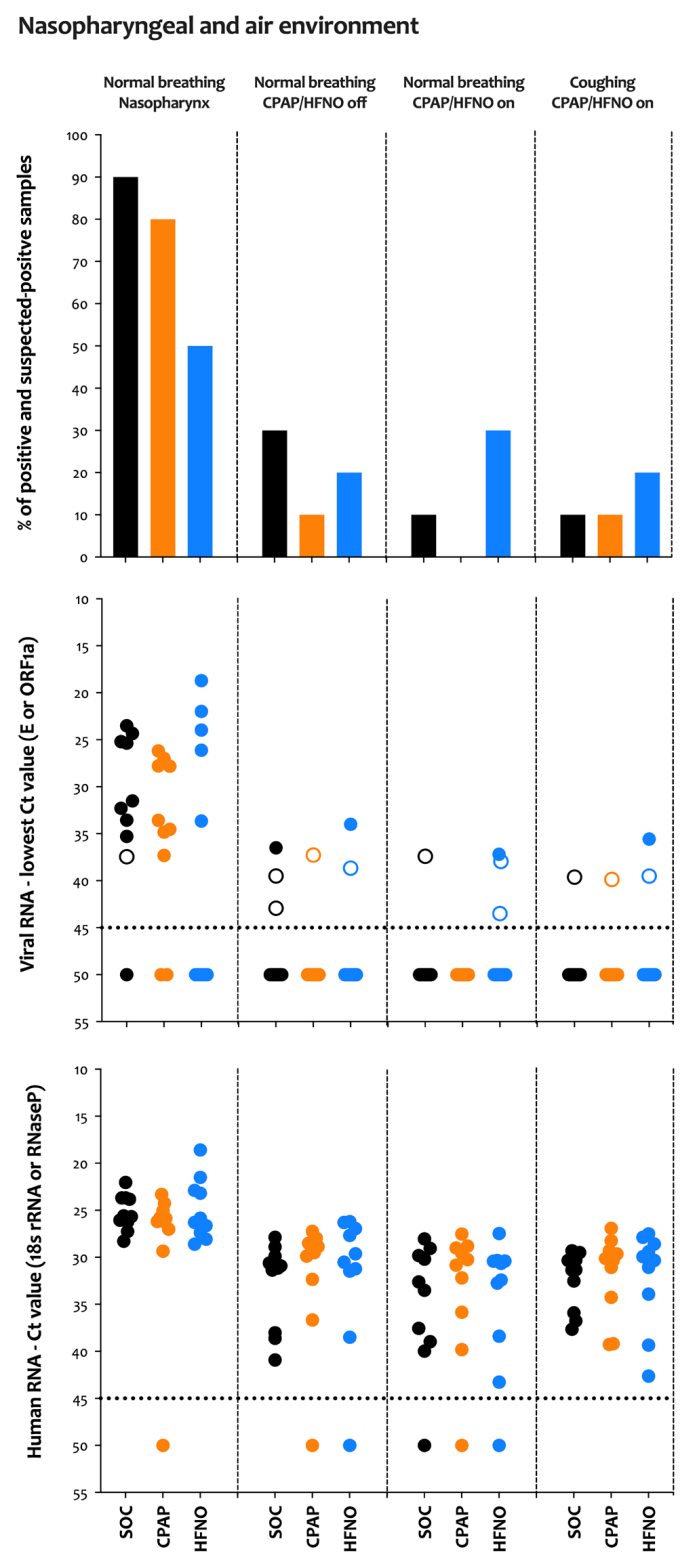
Viral and human RNA from nasopharyngeal and air samples. A total of three air samples were collected per participant. The first was at rest with the patient receiving supplementary oxygen via a face mask if able to tolerate a pause in CPAP/HFNO treatment for volunteers in these groups. The second sample was at rest with the CPAP/HFNO device on (if applicable). The third sample included voluntary coughing every 2 min with the CPAP/HFNO device on (if applicable). (Top) The proportion of samples that tested positive or suspected-positive for viral RNA. (Middle) Ct values for viral RNA. The dotted line signifies the detection threshold of 45; Ct values ≥45 were considered negative and were arbitrarily assigned a value of 50. Coloured circles show positive results (Ct value <45 in both E and ORF1a genes), whereas empty circles show suspected-positive results (a Ct value <45 in one of the two genes only). (Bottom) Ct values for human RNaseP in nasopharyngeal samples and human 18s rRNA in air samples. The dotted line signifies the detection threshold of 45; Ct values ≥45 were considered negative and arbitrarily assigned a value of 50. CPAP, continuous positive airway pressure; Ct, cycle time; HFNO, high-flow nasal oxygen; SOC, supplemental oxygen care.

Overall 9/30 (30%) of participants had at least one positive or suspected-positive result from one or more of the three air samples collected (figure 1, online supplemental figures 4 and 5). There were only 4/90 (4%) positive air samples, with an additional 10 suspected-positive. Furthermore, the Ct values for positive and suspected-positive air samples were substantially higher than paired samples in the nasopharynx, indicating minimal viral RNA in the air. The distribution of these positive and suspected-positive air samples did not indicate a relationship with the use of CPAP or coughing, but 7/14 (50%) of the positive and suspected-positive air samples were from the HFNO group despite only half of these participants testing positive for viral RNA on nasopharyngeal samples, although this was not statistically significant (table 2, figure 1). Human 18 s RNA was detectable in 85/90 (94%) of air samples. Again, the use of CPAP/HFNO and/or coughing did not appear to alter the quantity of human RNA. Post-hoc analyses explored potential differences between the nine participants who had tested positive or suspected-positive for viral RNA in one or more of the air samples, compared with the other 21 participants with negative air samples. Irrespective of the use of CPAP/HFNO at rest or on coughing, we found no significant differences with the environmental variables, days unwell at time of sampling, or nasopharyngeal Ct values between those who did and did not have viral RNA in air samples.

### Clinical surfaces were more contaminated with viral RNA than the air samples

**Figure 2:**
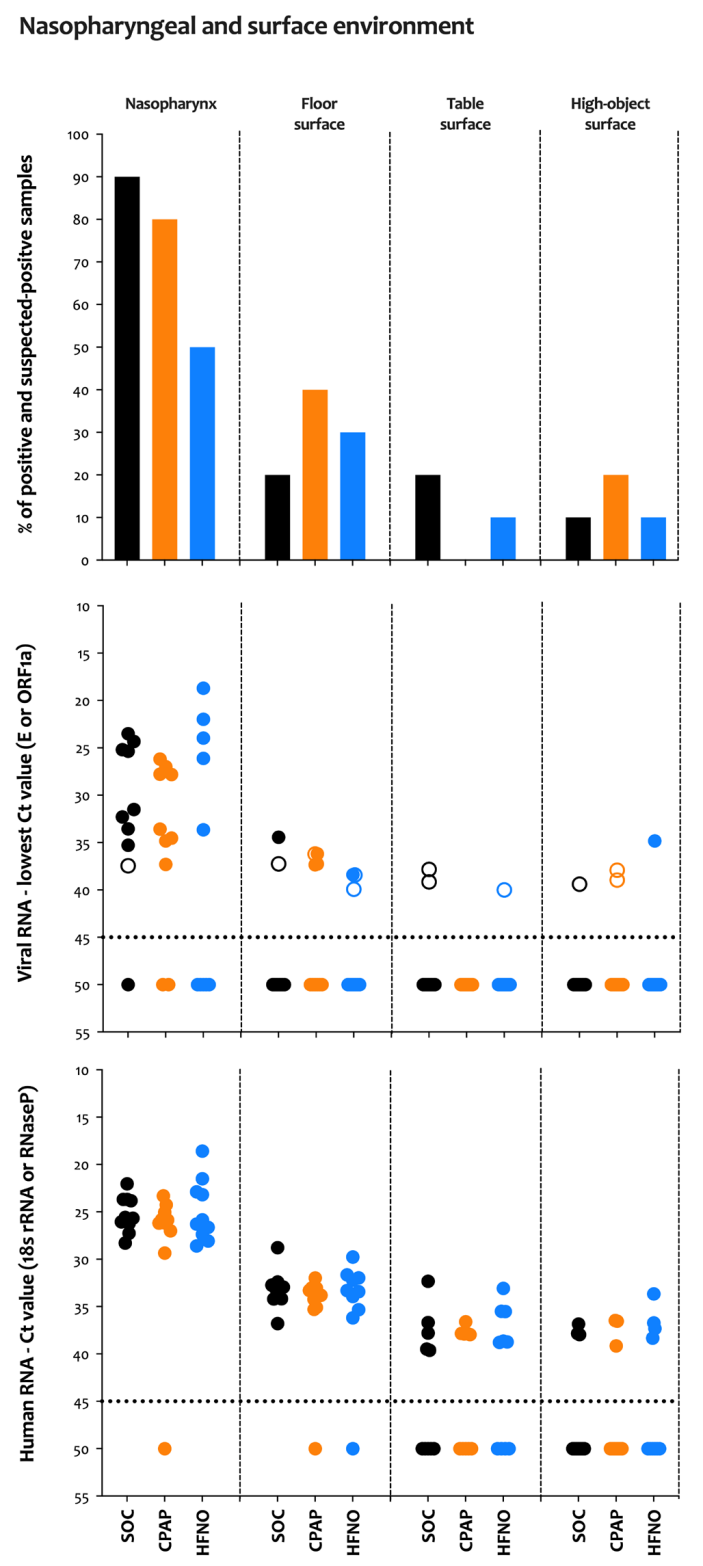
Viral and human RNA from surface samples. A total of three surface samples were collected per participant. The first was from the floor within 2 m of the bed, the second sample was from the bedside table at head height to the participant, and the third sample was from an object above participant head height (for example, a light fitting). (Top) The proportion of samples that tested positive or suspected-positive for viral RNA. (Middle) Ct values for viral RNA. The dotted line signifies the detection threshold of 45; Ct values ≥45 were considered negative and were arbitrarily assigned a value of 50. Coloured circles show positive results (Ct value <45 in both E and ORF1a genes), whereas empty circles show suspected-positive results (a Ct value <45 in one of the two genes only). (Bottom) Ct values for human RNaseP in surface samples. The dotted line signifies the detection threshold of 45; Ct values ≥45 were considered negative and were arbitrarily assigned a value of 50. CPAP, continuous positive airway pressure; Ct, cycle time; HFNO, high-flow nasal oxygen; SOC, supplemental oxygen care.

A higher proportion, 14/30 (47%), of participants had at least one positive or suspected-positive sample for viral RNA from one or more of the three surface samples collected (figure 2, online supplemental figures 6 and 7). Only four participants had a positive or suspected-positive sample in both an air and surface sample (two participants receiving supplemental oxygen and one from CPAP and HFNO). In total, 6/90 (7%) of surface swabs were positive for viral RNA; 5/30 (17%) floor samples tested positive (and four suspected-positive), no table surface samples tested positive (and three suspected-positive) and only one high-object surface sample tested positive (and three suspected-positives). As with our air samples, the Ct values for viral genes were greater than those recorded from the nasopharynx, and there were no differences with the use of CPAP/HFNO on any surface type. The floor was the most frequently contaminated surface (30%) followed by the high-object surfaces (13%) and tables (10%). Human RNA could be detected in 28/30 (93%) floor samples, 16/30 (53%) table samples and only 10/30 (33%) high-object surface samples. The Ct values for human RNaseP steadily increased from nasopharyngeal samples to floor, table and then the high-object samples. As before, the subset of participants with one or more positive or suspected-positive surface sample for viral RNA (n=14) were compared against participants who had negative surface swabs (n=16). The Ct values for viral RNA did not appear to vary significantly with the number of days unwell or nasopharyngeal Ct values between those who did and did not have viral RNA in surface samples. Lower room humidity was more common with positive surface samples and no significant differences were observed with other environmental measures.

### No viable virus could be recovered from any environmental sample that tested positive by PCR

In total, 51/210 (24%) samples were positive or suspected-positive for viral RNA and were cultured. Only one nasopharyngeal sample from a HFNO participant (E gene Ct 21.99) could demonstrate presence of viable (infective) virus and all other samples, including environmental samples, were negative. This individual had two positive air samples that had higher Ct values for viral RNA and were culture negative.

## Discussion

Our sampling study of the immediate environment of patients requiring non-invasive respiratory support for life-threatening COVID-19 disease found that few air and surface samples had measurable viral RNA contamination, irrespective of using CPAP/HFNO and/or coughing. Furthermore, the samples that did detect viral RNA by RT-qPCR, including those from the nasopharynx, failed to demonstrate biological viability in cell culture, except for one nasopharyngeal sample. These data question any significant additional risks to HCWs/other patients associated with the use of CPAP and HFNO, which are considered ‘aerosol-generating’, compared with the use of supplemental oxygen.

Consistent with other environmental sampling studies we found airborne and surface viral RNA contamination, 4% and 7% positive samples, respectively, within the vicinity of patients with COVID-19, although the degree of contamination was lower than that reported in most other studies.^12–20^ This was despite the majority of our participants having detectable viral RNA in the nasopharynx at time of sampling and irrespective of respiratory support type and/or coughing. Importantly, few previous studies included patients receiving non-invasive respiratory support, and from those studies that did there was little or no air contamination around non-invasive ventilation or HFNO.^16 18 19^ Furthermore, our findings concur with other studies that report surface contamination is not associated with mode of respiratory support including HFNO and/or non-invasive ventilation.^12 17^ Consistent with others we found a higher proportion of floor contamination than from other surfaces.^13 15^ This is unsurprising given the likely cumulative deposition of virus laden droplets from the air combined with potential transference of the virus from footwear. Heterogeneity between clinical setting, study design and methodology limits direct comparisons and is likely to account for the variation in findings between studies.

The lower degree of environmental contamination we found may be related to the stage of disease in our cohort of participants, with one sampling study reporting a decline in environmental contamination after the first week of illness.^13^ Participants in our study were, on average, in their second week of illness when admitted to hospital (mean 9 days) and when sampled (mean 12 days). SARS-CoV-2 viral shedding is at its highest quantity in early infection and the peak of infectivity coincides with symptom onset before a gradual decline to near the detection limit by day 21, although with significant individual variability.^32–35^ This kinetic is notably different from the related SARS-CoV-1 virus, where viral shedding peaks 7–10 days after symptom onset,^36 37^ and coincides more with the time when patients are admitted for hospital care. The SARS outbreak was associated with a high incidence of HCW and nosocomial transmission.^9^ Although we found no significant relationship between nasopharyngeal viral load and days of illness (or environmental contamination), patients with COVID-19 requiring non-invasive respiratory support are more likely to be at a stage of disease when it is plausible that host immunity has begun to establish control of viral shedding and infectivity.

The levels of environmental contamination in our study were not significantly influenced by CPAP/HFNO therapies and/or coughing. These findings broadly reflect data from aerosol-generation studies in healthy adult volunteers, which report that non-invasive positive pressure ventilation and HFNO did not generate significantly more aerosols (compared with other respiratory activities)^21 24^ or in fact reduced emissions for non-invasive positive pressure ventilation, HFNO^22^ and CPAP.^23^ This may be influenced by the semi-closed system of CPAP delivery and positive end expiratory pressures over the nose and mouth simultaneously, which limits aerosol/droplet dispersion from respiratory secretions. High-flow nasal cannulae to deliver HFNO leaves the mouth open for potential expulsion of infective secretions. Hamilton *et al* report that HFNO was associated with increased aerosol emission (flow rate and machine dependent), but this was generated by the machine, not the patient, hence unlikely to carry SARS-CoV-2 virus. Moreover, these studies consistently reported that the highest aerosol emissions were from coughing, irrespective of respiratory support modality, with at least a threefold increase.^21–23^ We did not find this signal in our data. However, these findings indicate that coughing is potentially the most hazardous source of infectious SARS-CoV-2 aerosols to HCWs and not the respiratory support device itself. The extrapolation of data from healthy volunteers may be limited to patients with COVID-19. However, one study has shown that the aerosol particle size distribution is similar between the two populations.^23^ Collectively, data from these studies and our own findings question whether the airborne mitigation measures are correctly aligned to the highest transmission risk, most likely from coughing and not the form of non-invasive respiratory support used.

Importantly, we found no biologically viable virus in cell culture from any positive or suspected-positive samples, except for one nasopharyngeal sample from a HFNO participant (E gene Ct value 21.99). This was a common finding from other environmental sampling studies that attempted culture.^12 14 16 18 20^ This may be due to air sampling methods, which are known to inactivate viruses and affect virus infectivity,^38 39^ although all of our surface and nasopharyngeal samples (except one) were also negative on cell culture. The stage of disease in our cohort of participants (mean 12 days of symptomatic illness at time of sampling) is likely to have influenced our findings, with one study demonstrating a median time of 7 days from symptom onset to viral clearance in culture, and the last positive culture being on day 12.^40^ Furthermore, lower Ct values have been correlated with a higher likelihood of successful culture,^35 41^ with studies demonstrating that viable virus could only be cultured from clinical samples and experimentally contaminated surfaces if the Ct value was <24 and <30, respectively.^16 42^ All our positive/suspected-positive environmental samples had a Ct value >30. This indicates that there was a poverty of viral RNA in the immediate environment of patients with COVID-19 receiving respiratory support therapies, and also that there was no detectable viable virus present as an infection risk to HCWs.

Our study has some notable strengths and limitations. Strengths include the ‘real-world’ setting, a standardised sampling strategy, concurrent air and surface sampling, collection of patient data and nasopharyngeal samples to understand the clinical context, and the use of human genetic material as a control. Finally, embedding the evaluation within the RECOVERY-Respiratory Support randomised controlled trial helped to minimise selection bias. Limitations include the lack of serial sampling, with findings representing a ‘snap shot’ picture, potential cross-contamination by other infected patients in cohorted areas, no particle size fractionation or concentration measurement (hence not able to differentiate between droplets and aerosols), air volume sampled only a small fraction of the total room air and potential air leaks from the sides of CPAP masks not being captured by the air sampler. Additionally, there are challenges in interpreting the significance of samples with low viral loads, and the extent to which PCR and viral culture technologies can be used as proxies for real-world infectivity remains uncertain. The small group sizes risk the study being underpowered with confounding chance observations, and larger studies are needed to develop the evidence needed to reliably inform pragmatic infection prevention control measures around the use of CPAP/HFNO.

## Conclusions

We found limited SARS-CoV-2 viral RNA within the immediate environment of hospitalised patients with COVID-19, and that this did not appear to be substantially influenced by the use of CPAP/HFNO devices or coughing, and importantly, no detectable biologically viable virus. This adds to the increasing evidence that for COVID-19, CPAP and HFNO may not be procedures with a higher transmission risk that are associated with their ‘aerosol generating’ classification. Rather, HCW exposure and nosocomial transmission may be more influenced by patient factors, such as coughing at earlier stages of infection, than the type of respiratory support used.

## Data Availability

All data relevant to the study are included in the article or uploaded as supplementary information.
