# Supplementary material for: SARS-CoV-2 environmental contamination from hospitalised patients with COVID-19 receiving aerosol-generating procedures
Source: Thorax. 2021 Nov 4;77(3):259–67. doi: 10.1136/thoraxjnl-2021-218035 (PMC8646974; doi:10.1136/thoraxjnl-2021-218035)
Supplement: Supplementary data [file thoraxjnl-2021-218035supp001.pdf]

Winslow *et al*; SARS-CoV-2 environmental contamination from hospitalised COVID-19 patients receiving aerosol-generating procedures.

SUPPLEMENTARY TABLES

Sample	Gene type		Treatment arm			Total	Exact Kruskal Wallis test
			SOC	CPAP	HFNO		P value
Nasopharyngeal swabs	E gene	N of positive value	9	8	5	22	0.088
		Mean	29.8	31.1	24.9	29.2	
		Standard deviation	5.3	4.4	5.6	5.4	
		Median	31.5	30.7	24	27.8	
		Interquartile range	25.2, 33.6	27.4, 34.7	22.0, 26.1	25.2, 33.7	
		Negative value	1	2	5	8	
	ORF1a gene	N	8	8	5	21	0.089
		Mean	30.2	32.2	26	29.9	
		Standard deviation	4.9	4.6	5.9	5.3	
		Median	30	31.5	25	28.3	
		Interquartile range	26.0, 34.3	28.1, 35.8	22.8, 27.0	26.1, 35.0	
		Negative value	2	2	5	9	
	RNaseP gene	N	10	9	10	29	0.855
		Mean	25.2	25.9	24.9	25.3	
		Standard deviation	1.9	1.7	3.2	2.4	
		Median	25.6	25.9	26.1	25.8	
		Interquartile range	23.7, 26.2	25.0, 26.2	22.9, 27.4	23.7, 26.6	
		Negative value	0	1	0	1	
Air sample (1st)	E gene	N	3	1	2	6	0.133
		Mean	40.6	37.3	37.7	39.1	
		Standard deviation	2	0	1.3	2.2	
		Median	39.5	37.3	37.7	39.1	
		Interquartile range	39.5, 42.9	37.3, 37.3	36.8, 38.7	37.3, 39.5	
		Negative value	7	9	8	24	
	ORF1a gene	N	1	0	1	2	n/a
		Mean	36.5	n/a	34	35.3	
		Standard deviation	0	n/a	0	1.8	
		Median	36.5	n/a	34	35.3	
		Interquartile range	36.5, 36.5	n/a	34.0, 34.0	34.0, 36.5	
		Negative value	9	10	9	28	
	18sRNA gene	N	10	9	9	28	0.131
		Mean	32.8	30	29.8	30.9	
		Standard deviation	4.6	2.9	3.9	4	
		Median	31	28.9	29.6	29.9	
		Interquartile range	29.9, 38.0	28.5, 29.9	27.0, 31.2	28.2, 31.4	
		Negative value	0	1	1	2	
Air sample (2nd)	E gene	N	1	0	3	4	n/a
		Mean	37.4	n/a	40.4	39.7	
		Standard deviation	0	n/a	2.8	2.8	

Winslow RL, *et al. Thorax* 2022; 77:259–267. doi: 10.1136/thoraxjnl-2021-218035

		Median	37.4	n/a	39.7	38.8	
		Interquartile range	37.4, 37.4	n/a	38.0, 43.5	37.7, 41.6	
		Negative value	9	10	7	26	
	ORF1a gene	N	0	0	1	1	n/a
		Mean	n/a	n/a	37.2	37.2	
		Standard deviation	n/a	n/a	0	0	
		Median	n/a	n/a	37.2	37.2	
		Interquartile range	n/a	n/a	37.2, 37.2	37.2, 37.2	
		Negative value	10	10	9	29	
	18sRNA gene	N	9	9	9	27	0.574
		Mean	33.3	31.5	32.9	32.6	
		Standard deviation	4.5	3.9	4.9	4.4	
		Median	32.6	30.2	30.7	30.7	
		Interquartile range	29.8, 37.6	29.0, 32.2	30.4, 32.8	29.6, 35.9	
		Negative value	1	1	1	3	
**Air sample (3rd)**	E gene	N	1	1	2	4	0.500
		Mean	39.6	39.9	38.5	39.1	
		Standard deviation	0	0	1.4	1.1	
		Median	39.6	39.9	38.5	39.6	
		Interquartile range	39.6, 39.6	39.9, 39.9	37.6, 39.5	38.5, 39.7	
		Negative value	9	9	8	26	
	ORF1a gene	N	0	0	1	1	n/a
		Mean	n/a	n/a	35.6	35.6	
		Standard deviation	n/a	n/a	0	0	
		Median	n/a	n/a	35.6	35.6	
		Interquartile range	n/a	n/a	35.6, 35.6	35.6, 35.6	
		Negative value	10	10	9	29	
	18sRNA gene	N	10	10	10	30	0.583
		Mean	32.5	31.8	32.1	32.1	
		Standard deviation	3.1	4.3	5.1	4.1	
		Median	31.3	30.2	30.1	30.3	
		Interquartile range	30.3, 35.9	29.3, 34.3	28.6, 33.9	29.4, 34.3	
		Negative value	0	0	0	0	
**Surface sample (1st)**	E gene	N	1	3	2	6	0.100
		Mean	34.4	36.9	39.2	37.3	
		Standard deviation	0	0.6	1.1	1.9	
		Median	34.4	37.3	39.2	37.3	
		Interquartile range	34.4, 34.4	36.2, 37.3	38.4, 39.9	36.2, 38.4	
		Negative value	9	7	8	24	
	ORF1a gene	N	2	4	2	8	0.210
		Mean	37.6	37.8	39.4	38.1	
		Standard deviation	0.4	1.2	1.4	1.2	
		Median	37.6	38	39.4	38	

Winslow RL, *et al. Thorax* 2022; 77:259–267. doi: 10.1136/thoraxjnl-2021-218035

		Interquartile range	37.3, 37.8	37.1, 38.5	38.4, 40.4	37.6, 38.7	
		Negative value	8	6	8	22	
	RNaseP gene	N	10	9	9	28	0.658
		Mean	33.2	33.7	33.1	33.3	
		Standard deviation	2	1	2	1.7	
		Median	33	33.8	33.3	33.3	
		Interquartile range	32.7, 34.2	33.1, 34.2	32.0, 33.9	32.6, 34.2	
		Negative value	0	1	1	2	
**Surface sample (2nd)**	E gene	N	0	0	1	1	n/a
		Mean	n/a	n/a	40	40	
		Standard deviation	n/a	n/a	0	0	
		Median	n/a	n/a	40	40	
		Interquartile range	n/a	n/a	40.0, 40.0	40.0, 40.0	
		Negative value	10	10	9	29	
	ORF1a gene	N	2	0	0	2	n/a
		Mean	38.5	n/a	n/a	38.5	
		Standard deviation	0.9	n/a	n/a	0.9	
		Median	38.5	n/a	n/a	38.5	
		Interquartile range	37.8, 39.2	n/a	n/a	37.8, 39.2	
		Negative value	8	10	10	28	
	RNaseP gene	N	5	5	6	16	0.947
		Mean	37.2	37.6	36.7	37.2	
		Standard deviation	3	0.6	2.4	2.1	
		Median	37.8	37.9	37.1	37.9	
		Interquartile range	36.7, 39.5	37.8, 37.9	35.5, 38.7	36.1, 38.7	
		Negative value	5	5	4	14	
**Surface sample (3rd)**	E gene	N	1	2	1	4	0.500
		Mean	39.4	38.4	34.8	37.8	
		Standard deviation	0	0.7	0	2.1	
		Median	39.4	38.4	34.8	38.4	
		Interquartile range	39.4, 39.4	37.9, 39.0	34.8, 34.8	36.4, 39.2	
		Negative value	9	8	9	26	
	ORF1a gene	N	0	0	1	1	n/a
		Mean	n/a	n/a	35.4	35.4	
		Standard deviation	n/a	n/a	0	0	
		Median	n/a	n/a	35.4	35.4	
		Interquartile range	n/a	n/a	35.4, 35.4	35.4, 35.4	
		Negative value	10	10	9	29	
	RNaseP gene	N	3	3	4	10	0.791
		Mean	37.6	37.4	36.5	37.1	
		Standard deviation	0.6	1.5	2	1.5	
		Median	37.9	36.6	37	37.1	
		Interquartile range	36.8, 38.0	36.5, 39.2	35.2, 37.8	36.6, 38.0	

Winslow RL, *et al. Thorax* 2022; 77:259–267. doi: 10.1136/thoraxjnl-2021-218035

		Negative value	7	7	6	20	

sTab. 1. Additional post-hoc analytical results for laboratory data.
In this analysis, all Ct values >45 were not included. Test is not applicable when number of observations is less than number of treatment groups or there is no valid observation in at least one arm. Overall difference across treatment arms was assessed using Exact Kruskal Wallis test. The interpretation from using this alternative statistical analysis were not different from those presented in the main paper. SOC, supplemental oxygen care. CPAP, continuous positive airway pressure. HFNO, high-flow nasal oxygen. n/a, not applicable.

SUPPLEMENTARY FIGURES

Baseline clinical features

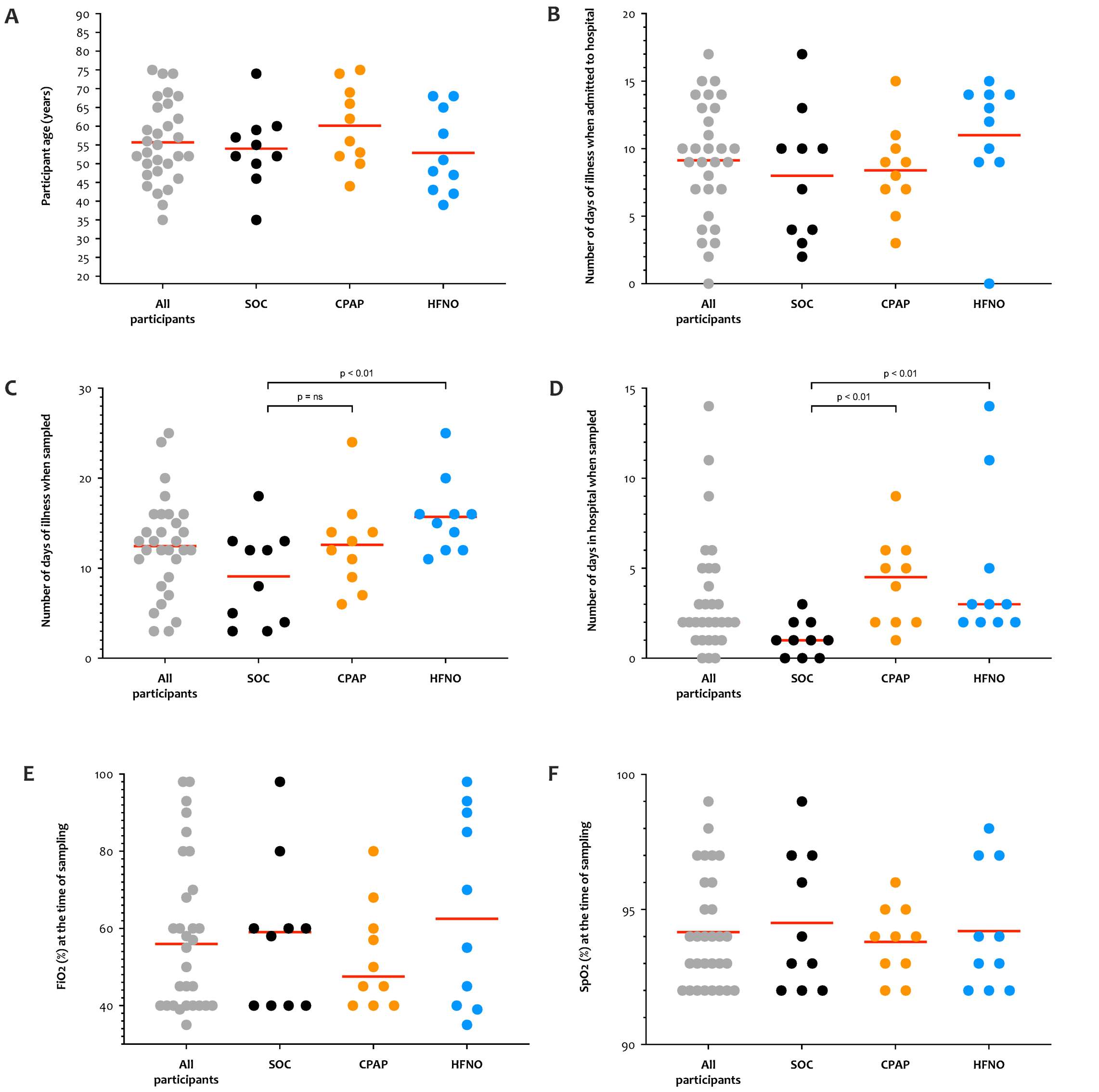

sFig. 1. Baseline clinical characteristics of the study population.
(A) Study participant ages at time of enrolment. *The red bar denotes the mean.* ***(B) The number of days with COVID-19 symptoms at the time of hospital admission****. The red bar denotes the mean.* ***(C) The number of days with COVID-19 symptoms at the time of sampling.*** *The red bar denotes the mean. HFNO participants were sampled having been unwell for longer than SOC participants (mean SOC 9.1 days vs mean HFNO 15.7 days, p=0.01, two-tailed unpaired t-test).* ***(D) The number of days with in hospital at the time of sampling.*** *The red bar denotes the median. The median duration of hospital stay for SOC participants was one day, which was significantly lower than both CPAP and HFNO participants (median 4.5 and 3 days respectively, p<0.01 by two-tailed Mann-Whitney tests for CPAP and HFNO compared with SOC).* ***(E) % FiO_2*** *and* ***(F) SpO_2*** *at the time of sampling which did not differ between study groups (red bar denotes the median and mean respectively). SOC, supplemental oxygen care. CPAP, continuous positive airway pressure. HFNO, high-flow nasal oxygen. FiO_2, fraction of inspired oxygen. SpO_2, oxygen saturation.*

Baseline environmental features

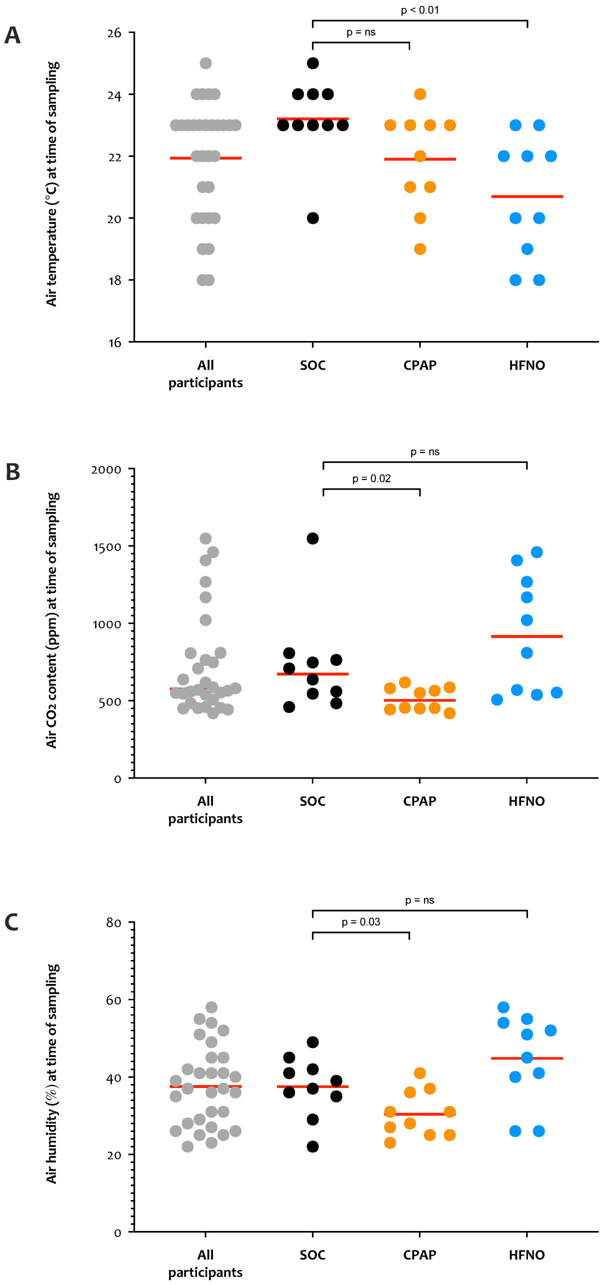

sFig. 2. Baseline environmental characteristics of the clinical environments.
(A) Room air temperature. *The red bar denotes the mean. Air temperature from HFNO participants was lower than SOC participants (mean SOC 23.2 ℃ vs mean HFNO 20.7 ℃, p<0.01, two-tailed unpaired t-test).* ***(B) Room air CO_2 content.*** *The red bar denotes the median. The median CO_2 content for SOC participants 673ppm, which was significantly higher than areas in use for CPAP (median 502ppm, p=0.02 by two-tailed Mann-Whitney tests).* ***(C) Room air humidity.*** *The red bar denotes the mean. The mean humidity of air around SOC participants was 37.6%, which was significantly higher than areas in use for CPAP (mean 30.4%, p=0.03 by two tailed unpaired t-test). SOC, supplemental oxygen care. CPAP, continuous positive airway pressure. HFNO, high-flow nasal oxygen.*

Correlation plots with nasal RNA CT values

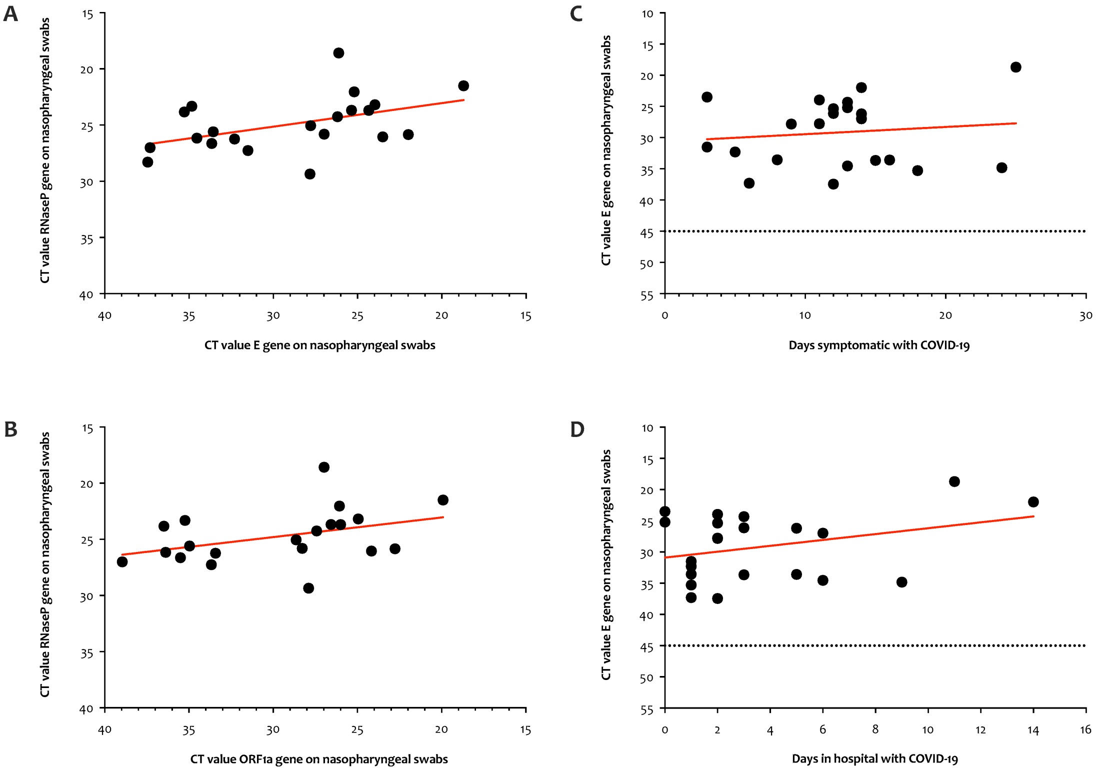

sFig. 3. Nasopharyngeal human and viral RNA exploratory correlation plots.
The red lines mark the linear regression analysis, dotted lines signify a Ct value of 45 that was used to define a negative result. ***(A) Human RNaseP Ct values vs viral E gene Ct values and (B) Viral ORF1a gene Ct values. (C) Viral E gene Ct values vs days unwell with COVID-19 symptoms and (D) Days in hospital.*** *There was only a weak statistically significant correlation between human RNaseP Ct values and the viral E gene Ct value (r^2=0.2, p=0.03, Pearson's correlation), but not with ORF1a (r^2=0.15, p=0.09, Pearson's correlation).*

Winslow *et al*; SARS-CoV-2 environmental contamination from hospitalised COVID-19 patients receiving aerosol-generating procedures.

Viral RNA from air samples by individuals

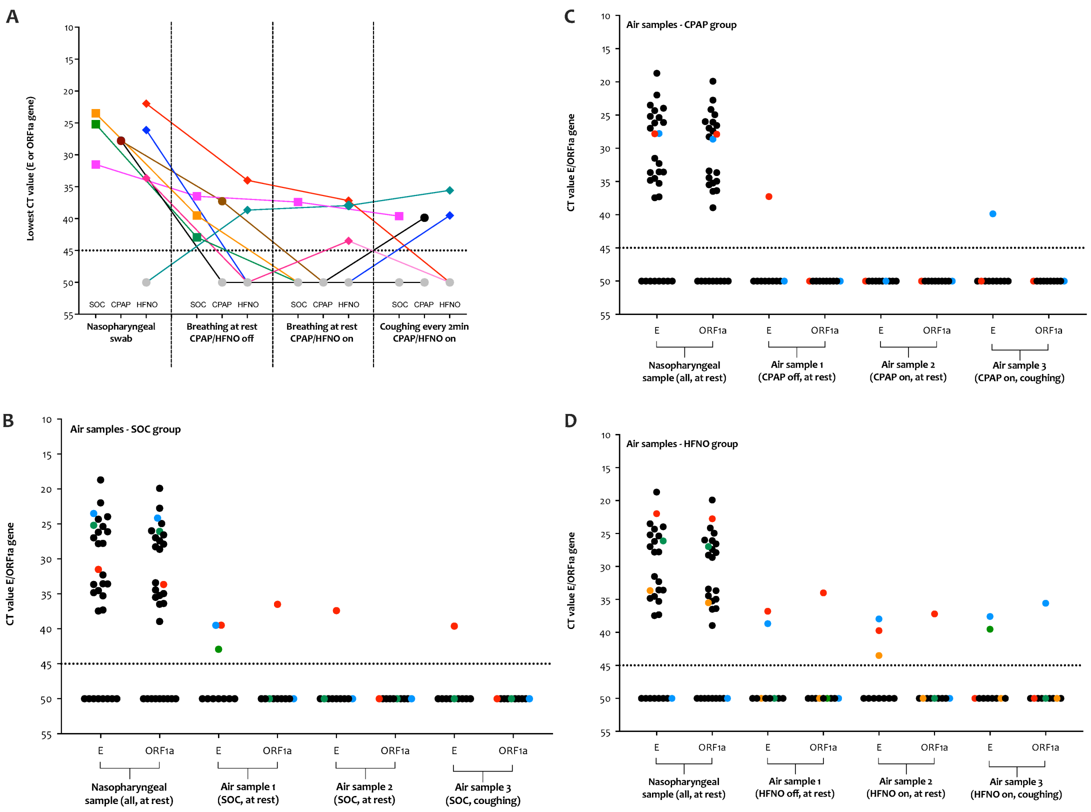

sFig. 4. Viral RNA results from the nasopharynx and air samples linked as individual participants.
The dotted lines signify a Ct value of 45 that was used to define a negative result. Ct values ≥45 were considered negative and were arbitrarily assigned a value of 50. ***(A) All individuals linked from having had at least one positive or suspected-positive air sample.*** *The connected coloured dots are used to identify each participant with at least one positive Ct value for E/ORF1a genes (all study groups combined).* ***(B) SOC participants, (C) CPAP participants*** *and* ***(D) HFNO participants.*** *The coloured dots are used to identify each participant with at least one positive Ct value for E/ORF1a genes. SOC, supplemental oxygen care. CPAP, continuous positive airway pressure. HFNO, high-flow nasal oxygen.*

Winslow RL, *et al. Thorax* 2022; 77:259–267. doi: 10.1136/thoraxjnl-2021-218035

Winslow *et al*; SARS-CoV-2 environmental contamination from hospitalised COVID-19 patients receiving aerosol-generating procedures.

Comparisons between individuals with positive and negative air samples

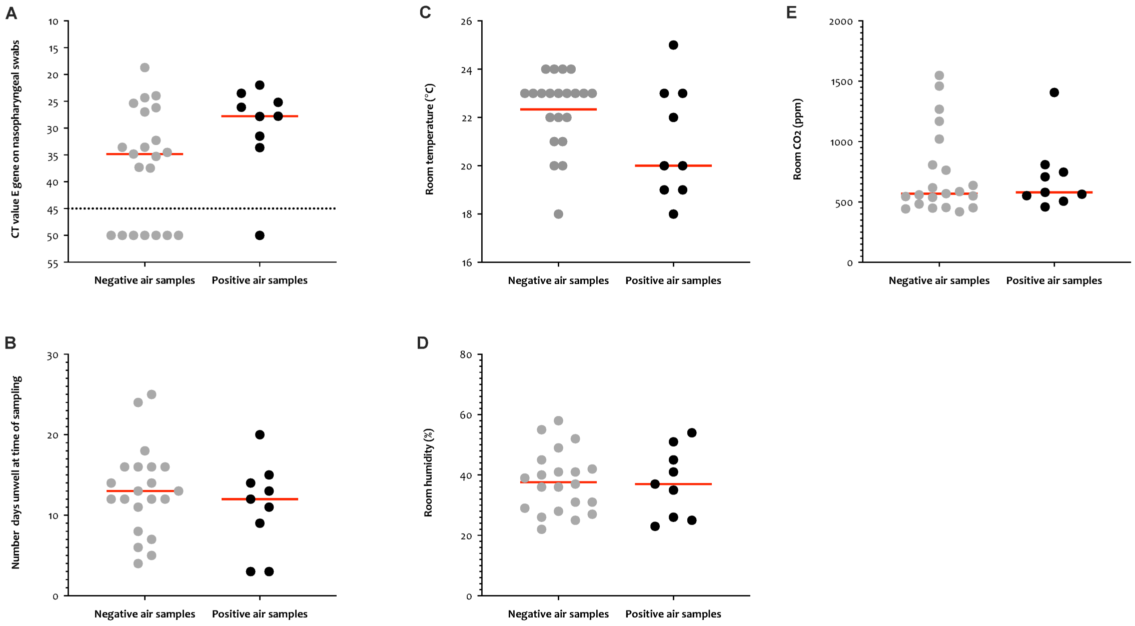

sFig. 5. Sub-set comparative analysis of participants with positive and negative air samples.
The red lines mark the mean or median Ct value as appropriate. Any single positive or suspected-positive air samples was used to identify the participants has having 'positive air samples' (n=9). The two sub-populations of participants were then compared for differences in the ***(A) Ct values in the E gene from nasopharyngeal samples****. The dotted lines signify a Ct value of 45 that was used to define a negative result.* ***(B) Number of days unwell with COVID-19 symptoms*** *and environmental factors of* ***(C) Room air temperature, (D) Room air humidity*** *and* ***(E) Room air CO_2 content****. Comparative testing (unpaired t-tests or Mann-Whitney tests, as appropriate) found no statistically significant differences between each of these sub-populations for any conditions shown above.*

Winslow RL, *et al. Thorax* 2022; 77:259–267. doi: 10.1136/thoraxjnl-2021-218035

BMJ Publishing Group Limited (BMJ) disclaims all liability and responsibility arising from any reliance placed on this supplemental material which has been supplied by the author(s)

Viral RNA from surface samples by individuals

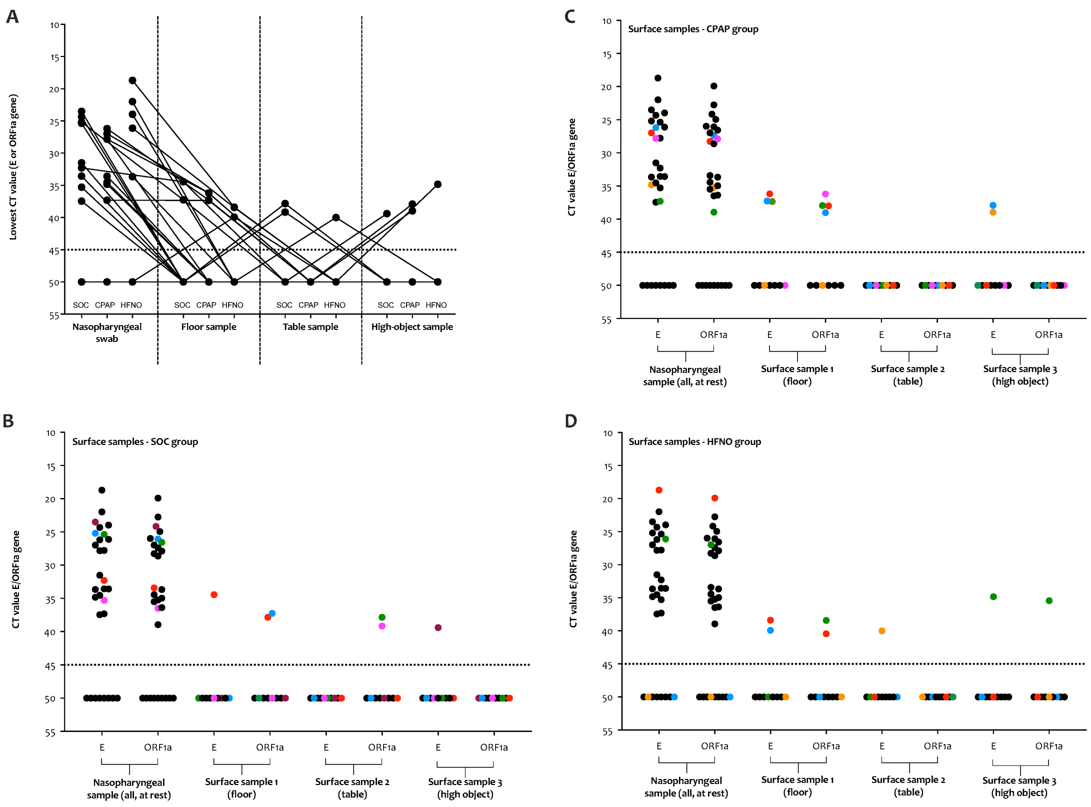

sFig. 6. Viral RNA results from the nasopharynx and surface samples linked as individual participants. *The dotted lines signify a Ct value of 45 that was used to define a negative result. Ct values ≥45 were considered negative and were arbitrarily assigned a value of 50.* ***(A) All individuals linked from having had at least one positive or suspected-positive surface sample.*** *The connected dots are used to identify each participant with at least one positive Ct value for E/ORF1a genes (all study groups combined).* ***(B) SOC participants, (C) CPAP participants*** *and* ***(D) HFNO participants.*** *The coloured dots are used to identify each participant with at least one positive Ct value for E/ORF1a genes. SOC, supplemental oxygen care. CPAP, continuous positive airway pressure. HFNO, high-flow nasal oxygen.*

Winslow RL, *et al. Thorax* 2022; 77:259–267. doi: 10.1136/thoraxjnl-2021-218035

Winslow *et al*; SARS-CoV-2 environmental contamination from hospitalised COVID-19 patients receiving aerosol-generating procedures.

Comparisons between individuals with positive and negative surface samples

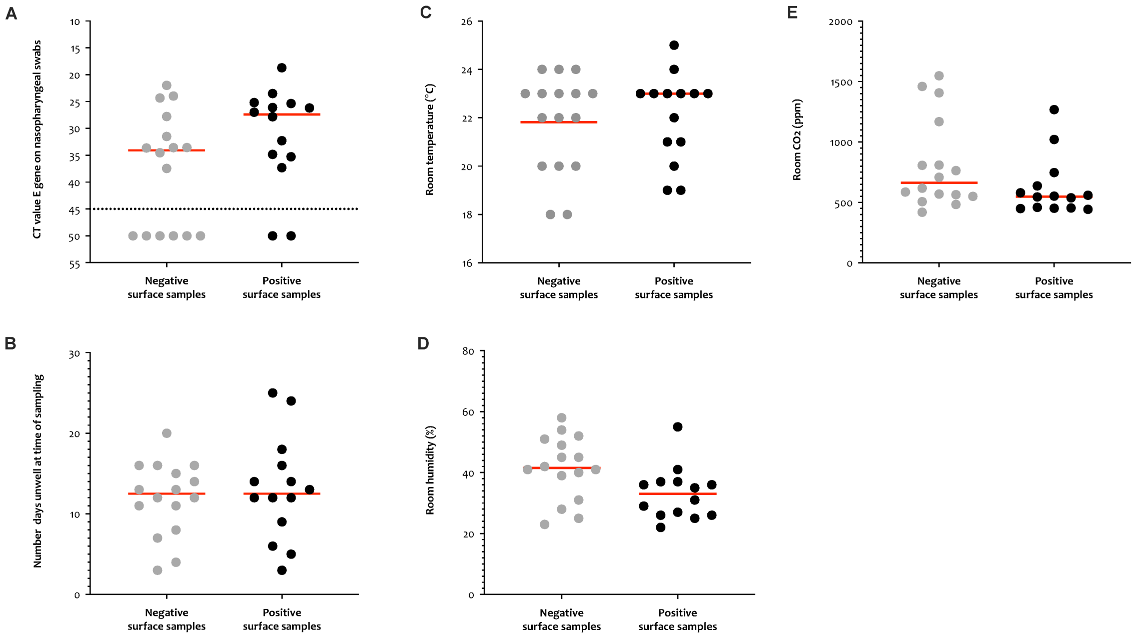

sFig. 7. Sub-set comparative analysis of participants with positive and negative surface samples.
The red lines mark the mean or median Ct value as appropriate. Any single positive or suspected-positive surface sample was used to identify the participants has having 'positive surface samples' (n=14). The two sub-populations of participants were then compared for differences in the ***(A) Ct values in the E gene from nasopharyngeal samples****. The dotted lines signify a Ct value of 45 that was used to define a negative result.* ***(B) Number of days unwell with COVID-19 symptoms*** *and environmental factors of* ***(C) Room air temperature, (D) Room air humidity*** *and* ***(E) Room air CO_2 content****. Comparative testing (unpaired t-tests or Mann-Whitney tests, as appropriate) found no statistically significant differences between each of these sub-populations for any conditions shown above, with the exception of room air humidity (p=0.02, two-tailed unpaired t-test). For participants in the positive/suspected-positive group for surface samples there was only a weak and non-significant correlation between room air humidity and the Ct value for viral E/ORF1a genes (r^2=0.41, p=0.14, two-tailed Pearson's correlation).*
